# Supplementary material for: Resolving the full spectrum of human genome variation using Linked-Reads
Source: Genome Res. 2019 Apr;29(4):635–45. doi: 10.1101/gr.234443.118 (PMC6442396; doi:10.1101/gr.234443.118)
Supplement: Supplemental Material [file supp_gr.234443.118_Supplemental_longranger-2.2.zip › longranger-2.2.tar.bz2!/lr2.2-public/lib/stan/cmdstan/stan/doc/api/html/classstan_1_1mcmc_1_1base__mcmc.html]

Stan: stan::mcmc::base\_mcmc Class Reference


|  |  |
| --- | --- |
|  | Stan  2.5.0  probability, sampling & optimization |


- Main Page
- Related Pages
- Namespaces
- Classes
- Files

- Class List
- Class Index
- Class Hierarchy
- Class Members

All Classes Namespaces Files Functions Variables Typedefs Enumerations Enumerator Friends Macros Pages

- stan
- mcmc
- base\_mcmc

Public Member Functions |
Protected Attributes |
List of all members

stan::mcmc::base\_mcmc Class Referenceabstract

`#include <base_mcmc.hpp>`

Inheritance diagram for stan::mcmc::base\_mcmc:

|  |  |
| --- | --- |
| Public Member Functions | |
|  | base\_mcmc (std::ostream \*o, std::ostream \*e) |
|  | |
| virtual | ~base\_mcmc () |
|  | |
| virtual sample | transition (sample &init\_sample)=0 |
|  | |
| std::string | name () |
|  | |
| virtual void | write\_sampler\_param\_names (std::ostream &o) |
|  | |
| virtual void | write\_sampler\_params (std::ostream &o) |
|  | |
| virtual void | get\_sampler\_param\_names (std::vector< std::string > &names) |
|  | |
| virtual void | get\_sampler\_params (std::vector< double > &values) |
|  | |
| virtual void | write\_sampler\_state (std::ostream \*o) |
|  | |
| virtual void | get\_sampler\_diagnostic\_names (std::vector< std::string > &model\_names, std::vector< std::string > &names) |
|  | |
| virtual void | get\_sampler\_diagnostics (std::vector< double > &values) |
|  | |

|  |  |
| --- | --- |
| Protected Attributes | |
| std::string | name\_ |
|  | |
| std::ostream \* | out\_stream\_ |
|  | |
| std::ostream \* | err\_stream\_ |
|  | |

## Detailed Description

Definition at line 13 of file base\_mcmc.hpp.

## Constructor & Destructor Documentation

|  |  |  |  |
| --- | --- | --- | --- |
| stan::mcmc::base\_mcmc::base\_mcmc | ( | std::ostream \* | *o*, |
|  |  | std::ostream \* | *e* |
|  | ) |  |  |

inline

Definition at line 17 of file base\_mcmc.hpp.

|  |  |  |  |  |
| --- | --- | --- | --- | --- |
| virtual stan::mcmc::base\_mcmc::~base\_mcmc | ( |  | ) |  |

inlinevirtual

Definition at line 19 of file base\_mcmc.hpp.

## Member Function Documentation

|  |  |  |  |
| --- | --- | --- | --- |
| virtual void stan::mcmc::base\_mcmc::get\_sampler\_diagnostic\_names | ( | std::vector< std::string > & | *model\_names*, |
|  |  | std::vector< std::string > & | *names* |
|  | ) |  |  |

inlinevirtual

Reimplemented in stan::mcmc::base\_hmc< M, P, H, I, BaseRNG >, stan::mcmc::base\_hmc< M, diag\_e\_point, diag\_e\_metric, expl\_leapfrog, BaseRNG >, stan::mcmc::base\_hmc< M, dense\_e\_point, dense\_e\_metric, expl\_leapfrog, BaseRNG >, and stan::mcmc::base\_hmc< M, unit\_e\_point, unit\_e\_metric, expl\_leapfrog, BaseRNG >.

Definition at line 35 of file base\_mcmc.hpp.

|  |  |  |  |  |  |
| --- | --- | --- | --- | --- | --- |
| virtual void stan::mcmc::base\_mcmc::get\_sampler\_diagnostics | ( | std::vector< double > & | *values* | ) |  |

inlinevirtual

Reimplemented in stan::mcmc::base\_hmc< M, P, H, I, BaseRNG >, stan::mcmc::base\_hmc< M, diag\_e\_point, diag\_e\_metric, expl\_leapfrog, BaseRNG >, stan::mcmc::base\_hmc< M, dense\_e\_point, dense\_e\_metric, expl\_leapfrog, BaseRNG >, and stan::mcmc::base\_hmc< M, unit\_e\_point, unit\_e\_metric, expl\_leapfrog, BaseRNG >.

Definition at line 38 of file base\_mcmc.hpp.

|  |  |  |  |  |  |
| --- | --- | --- | --- | --- | --- |
| virtual void stan::mcmc::base\_mcmc::get\_sampler\_param\_names | ( | std::vector< std::string > & | *names* | ) |  |

inlinevirtual

Reimplemented in stan::mcmc::base\_nuts< M, P, H, I, BaseRNG >, stan::mcmc::base\_nuts< M, diag\_e\_point, diag\_e\_metric, expl\_leapfrog, BaseRNG >, stan::mcmc::base\_nuts< M, dense\_e\_point, dense\_e\_metric, expl\_leapfrog, BaseRNG >, stan::mcmc::base\_nuts< M, unit\_e\_point, unit\_e\_metric, expl\_leapfrog, BaseRNG >, stan::mcmc::base\_static\_hmc< M, P, H, I, BaseRNG >, stan::mcmc::base\_static\_hmc< M, diag\_e\_point, diag\_e\_metric, expl\_leapfrog, BaseRNG >, stan::mcmc::base\_static\_hmc< M, dense\_e\_point, dense\_e\_metric, expl\_leapfrog, BaseRNG >, and stan::mcmc::base\_static\_hmc< M, unit\_e\_point, unit\_e\_metric, expl\_leapfrog, BaseRNG >.

Definition at line 29 of file base\_mcmc.hpp.

|  |  |  |  |  |  |
| --- | --- | --- | --- | --- | --- |
| virtual void stan::mcmc::base\_mcmc::get\_sampler\_params | ( | std::vector< double > & | *values* | ) |  |

inlinevirtual

Reimplemented in stan::mcmc::base\_nuts< M, P, H, I, BaseRNG >, stan::mcmc::base\_nuts< M, diag\_e\_point, diag\_e\_metric, expl\_leapfrog, BaseRNG >, stan::mcmc::base\_nuts< M, dense\_e\_point, dense\_e\_metric, expl\_leapfrog, BaseRNG >, stan::mcmc::base\_nuts< M, unit\_e\_point, unit\_e\_metric, expl\_leapfrog, BaseRNG >, stan::mcmc::base\_static\_hmc< M, P, H, I, BaseRNG >, stan::mcmc::base\_static\_hmc< M, diag\_e\_point, diag\_e\_metric, expl\_leapfrog, BaseRNG >, stan::mcmc::base\_static\_hmc< M, dense\_e\_point, dense\_e\_metric, expl\_leapfrog, BaseRNG >, and stan::mcmc::base\_static\_hmc< M, unit\_e\_point, unit\_e\_metric, expl\_leapfrog, BaseRNG >.

Definition at line 31 of file base\_mcmc.hpp.

|  |  |  |  |  |
| --- | --- | --- | --- | --- |
| std::string stan::mcmc::base\_mcmc::name | ( |  | ) |  |

inline

Definition at line 23 of file base\_mcmc.hpp.

|  |  |  |  |  |  |
| --- | --- | --- | --- | --- | --- |
| virtual sample stan::mcmc::base\_mcmc::transition | ( | sample & | *init\_sample* | ) |  |

pure virtual

Implemented in stan::mcmc::base\_nuts< M, P, H, I, BaseRNG >, stan::mcmc::base\_nuts< M, diag\_e\_point, diag\_e\_metric, expl\_leapfrog, BaseRNG >, stan::mcmc::base\_nuts< M, dense\_e\_point, dense\_e\_metric, expl\_leapfrog, BaseRNG >, stan::mcmc::base\_nuts< M, unit\_e\_point, unit\_e\_metric, expl\_leapfrog, BaseRNG >, stan::mcmc::adapt\_dense\_e\_static\_hmc< M, BaseRNG >, stan::mcmc::adapt\_diag\_e\_static\_hmc< M, BaseRNG >, stan::mcmc::adapt\_dense\_e\_nuts< M, BaseRNG >, stan::mcmc::adapt\_diag\_e\_nuts< M, BaseRNG >, stan::mcmc::adapt\_unit\_e\_static\_hmc< M, BaseRNG >, stan::mcmc::base\_static\_hmc< M, P, H, I, BaseRNG >, stan::mcmc::base\_static\_hmc< M, diag\_e\_point, diag\_e\_metric, expl\_leapfrog, BaseRNG >, stan::mcmc::base\_static\_hmc< M, dense\_e\_point, dense\_e\_metric, expl\_leapfrog, BaseRNG >, stan::mcmc::base\_static\_hmc< M, unit\_e\_point, unit\_e\_metric, expl\_leapfrog, BaseRNG >, stan::mcmc::adapt\_unit\_e\_nuts< M, BaseRNG >, and stan::mcmc::fixed\_param\_sampler.

|  |  |  |  |  |  |
| --- | --- | --- | --- | --- | --- |
| virtual void stan::mcmc::base\_mcmc::write\_sampler\_param\_names | ( | std::ostream & | *o* | ) |  |

inlinevirtual

Reimplemented in stan::mcmc::base\_nuts< M, P, H, I, BaseRNG >, stan::mcmc::base\_nuts< M, diag\_e\_point, diag\_e\_metric, expl\_leapfrog, BaseRNG >, stan::mcmc::base\_nuts< M, dense\_e\_point, dense\_e\_metric, expl\_leapfrog, BaseRNG >, stan::mcmc::base\_nuts< M, unit\_e\_point, unit\_e\_metric, expl\_leapfrog, BaseRNG >, stan::mcmc::base\_static\_hmc< M, P, H, I, BaseRNG >, stan::mcmc::base\_static\_hmc< M, diag\_e\_point, diag\_e\_metric, expl\_leapfrog, BaseRNG >, stan::mcmc::base\_static\_hmc< M, dense\_e\_point, dense\_e\_metric, expl\_leapfrog, BaseRNG >, and stan::mcmc::base\_static\_hmc< M, unit\_e\_point, unit\_e\_metric, expl\_leapfrog, BaseRNG >.

Definition at line 25 of file base\_mcmc.hpp.

|  |  |  |  |  |  |
| --- | --- | --- | --- | --- | --- |
| virtual void stan::mcmc::base\_mcmc::write\_sampler\_params | ( | std::ostream & | *o* | ) |  |

inlinevirtual

Reimplemented in stan::mcmc::base\_nuts< M, P, H, I, BaseRNG >, stan::mcmc::base\_nuts< M, diag\_e\_point, diag\_e\_metric, expl\_leapfrog, BaseRNG >, stan::mcmc::base\_nuts< M, dense\_e\_point, dense\_e\_metric, expl\_leapfrog, BaseRNG >, stan::mcmc::base\_nuts< M, unit\_e\_point, unit\_e\_metric, expl\_leapfrog, BaseRNG >, stan::mcmc::base\_static\_hmc< M, P, H, I, BaseRNG >, stan::mcmc::base\_static\_hmc< M, diag\_e\_point, diag\_e\_metric, expl\_leapfrog, BaseRNG >, stan::mcmc::base\_static\_hmc< M, dense\_e\_point, dense\_e\_metric, expl\_leapfrog, BaseRNG >, and stan::mcmc::base\_static\_hmc< M, unit\_e\_point, unit\_e\_metric, expl\_leapfrog, BaseRNG >.

Definition at line 27 of file base\_mcmc.hpp.

|  |  |  |  |  |  |
| --- | --- | --- | --- | --- | --- |
| virtual void stan::mcmc::base\_mcmc::write\_sampler\_state | ( | std::ostream \* | *o* | ) |  |

inlinevirtual

Reimplemented in stan::mcmc::base\_hmc< M, P, H, I, BaseRNG >, stan::mcmc::base\_hmc< M, diag\_e\_point, diag\_e\_metric, expl\_leapfrog, BaseRNG >, stan::mcmc::base\_hmc< M, dense\_e\_point, dense\_e\_metric, expl\_leapfrog, BaseRNG >, and stan::mcmc::base\_hmc< M, unit\_e\_point, unit\_e\_metric, expl\_leapfrog, BaseRNG >.

Definition at line 33 of file base\_mcmc.hpp.

## Member Data Documentation

std::ostream\* stan::mcmc::base\_mcmc::err\_stream\_

protected

Definition at line 45 of file base\_mcmc.hpp.

std::string stan::mcmc::base\_mcmc::name\_

protected

Definition at line 38 of file base\_mcmc.hpp.

std::ostream\* stan::mcmc::base\_mcmc::out\_stream\_

protected

Definition at line 44 of file base\_mcmc.hpp.

---

The documentation for this class was generated from the following file:

- src/stan/mcmc/base\_mcmc.hpp


---

[ Stan Home Page ]

*© 2011–2014,
Stan Development Team.*
